# A systematic review of mathematical and machine learning models of Avian Influenza

**DOI:** 10.1016/j.onehlt.2025.101203

**Published:** 2025-09-17

**Authors:** Shixun Huang, Nicola Luigi Bragazzi, Zahra Movahedi Nia, Murray Gillies, Emma Gardner, Doris Leung, Itlala Gizo, Jude D. Kong

**Affiliations:** a Artificial Intelligence & Mathematical Modeling Lab (AIMM Lab), Dalla Lana School of Public Health, University of Toronto, 155 College St, Room 500, Toronto, ON M5T 3M7, Canada; b Department of Computer Science, University of Toronto, 40 St George St, Toronto, ON M5S 2E4, Canada; c Department of Food and Drugs, University of Parma, 43125 Parma, Italy; d Global South Artificial Intelligence for Pandemic and Epidemic Preparedness and Response Network (AI4PEP), Toronto, ON, Canada; e Africa-Canada Artificial Intelligence and Data Innovation Consortium (ACADIC), Toronto, ON, Canada; f Department of Mathematics, York University, Toronto, ON M3J 1P3, Canada; g Canada Animal Health Surveillance System (CAHSS), Animal Health Canada, Elora, ON N0B 1S0, Canada; h World Health Organization (WHO), Avenue Appia, Geneva 27, CH-1211, Switzerland; i World Organization for Animal Health (WOAH), 12, rue de Prony, 75017 Paris, France; j Institute of Health Policy, Management and Evaluation (IHPME), University of Toronto, Toronto, ON, Canada; k Department of Mathematics, University of Toronto, Bahen Centre for Information Technology, Room 6291, 40 St. George Street, Toronto, ON M5S 2E4, Canada

**Keywords:** Avian Influenza, Mathematical modeling, Machine learning, Hybrid models, Epidemiological modeling, Outbreak prediction, Risk assessment, Transmission dynamics, Compartmental models, Statistical models

## Abstract

Avian influenza (AI) is a highly transmissible disease with significant implications for public health, agriculture, and global food security. Mathematical, statistical, and machine learning-based models play a crucial role in understanding AI dynamics, predicting outbreaks, and evaluating intervention strategies. This systematic review assesses existing modeling approaches, categorizing studies into mathematical and statistical models, machine learning-based models, and hybrid models, with a focus on their applications in risk assessment, outbreak prediction, dynamic modeling, and parameter estimation. Following the PRISMA guidelines, a comprehensive literature search was conducted in PubMed/MEDLINE, Scopus, Web of Science, and Embase. The search strategy included machine learning-related terms combined with modeling approaches such as compartmental models (e.g., SEIR, SIR), statistical methods, machine learning algorithms (e.g., SVM, Random Forest, XGBoost), and hybrid frameworks. A total of 43 studies met the inclusion criteria: 26 (60.47 %) used mathematical/statistical models, 12 (27.91 %) used machine learning models, and 5 (11.63 %) employed hybrid models. Among mathematical/statistical models, 50 % addressed transmission dynamics, while machine learning models primarily focused on risk assessment (50 %) and outbreak prediction (41.67 %). Hybrid models, though less prevalent, contributed to enhanced prediction accuracy and understanding of transmission. However, validation remains inconsistent, with 25.58 % of mathematical/statistical models lacking explicit validation. Sensitivity analysis and numerical simulations dominate mathematical and statistical model validation, whereas machine learning studies commonly use F1-score, confusion matrices, and external validation datasets. Persistent challenges include limited generalizability of datasets, inconsistency in validation protocols, and high computational costs. This review highlights the need for enhanced data sharing, integration of environmental and real-time information, standardized validation methods, and further development of hybrid approaches to strengthen model reliability and improve the prediction and control of future AI outbreaks.

## 1 Introduction

Avian influenza, commonly known as bird flu, is an infectious disease caused by the influenza A virus. It was first identified in 1878 by researcher Edoardo Perroncito, who described it as a high mortality rate disease among chickens in Italy [1]. Avian influenza originates in waterfowl, which serve as asymptomatic natural reservoirs, can spread to poultry where it causes high morbidity and mortality, and may cross the species barrier to infect humans, leading to illnesses ranging from mild respiratory symptoms to severe, potentially fatal disease [2]. The first human case of avian influenza was detected in Hong Kong in 1997 and since then, sporadic human cases have been reported globally, all resulting from spillover events from infected animals or animal products, with no confirmed sustained human-to-human transmission [3]. This spread has posed significant epidemiological, ecological, and economic challenges, characterized by high mortality rates and rapid transmission, and has resulted in the death of millions of domestic and wild birds and mammals.

The global spread of avian influenza, particularly strains like H5N1 and H7N9, has raised urgent concerns due to the potential for large-scale outbreaks in both bird and mammal populations, including humans [4]. The high pathogenicity of certain viral strains, along with their ability to mutate and occasionally transmit to humans, presents a significant pandemic threat. For instance, viruses such as Ebola, Marburg, Lassa fever, Nipah, and coronaviruses (including SARS and MERS) have demonstrated epidemic and pandemic potential [5]. Furthermore, the rapid transmission among birds, especially in densely populated poultry farms, increases the risk of significant economic losses in the agriculture sector and endangers global food security [6]. The continued evolution of the virus and sporadic zoonotic transmission have made it an international public health priority, calling for more sophisticated tools to predict, monitor, and control its spread.

Mathematical, statistical, and machine learning-based models can play a crucial role in the surveillance and control of avian influenza. Such models can illustrate how the disease spreads within populations, allow for the simulation of various interventions to assess their potential effectiveness, and identify patterns to predict future outbreaks [7]. Given the ongoing threat posed by avian influenza and the potential for new, more virulent strains to emerge, there is an urgent need to synthesize existing modeling efforts to provide a clearer picture of how the disease can be controlled and mitigated.

This systematic review aims to categorize existing avian influenza models by major tasks, summarize the datasets they employ, review and compare their validation methods, and identify research gaps and future directions. By addressing these aims, the review enhances understanding of avian influenza dynamics and provides evidence to guide future research and policy decisions.

## 2 Methods

The methodology employed follows the Preferred Reporting Items for Systematic Reviews and Meta-Analyses (PRISMA) [8]. The PRISMA framework comprises three stages: Identification, Screening, and Eligibility.

### 2.1 Identification

In the Identification stage, a comprehensive and systematic search of the literature was conducted across multiple electronic databases, including PubMed/MEDLINE, Scopus, Web of Science, and Embase. These databases were selected for their extensive coverage of scientific, biomedical, and computational research. To capture a broad range of studies relevant to avian influenza modeling, a well-defined search string was used. The search string combined terms for avian influenza with a variety of modeling approaches: (“avian influenza” OR “bird flu” OR “bird influenza”) AND (“mathematical model*” OR “computational simulation*” OR “modeling stud*” OR “modelling stud*” OR “scenario analysis” OR “geospatial model*” OR “spatial model*” OR “ecological model*” OR “compartmentalized model*” OR “transmission dynamic model*” OR “epidemic model*” OR “machine learning” OR “artificial intelligence” OR “statistical model*” OR “agent-based model*” OR “in-host model*” OR “support-vector machine*” OR “random forest*” OR “decision tree*” OR “deep learning” OR “neural network*” OR XGBoost OR Adaboost OR “gradient boosting” OR LASSO OR elasticnet OR “ridge regression*” OR “penalized regression*” OR “regularized regression*”).

This search string was designed to encompass a wide variety of modeling approaches, including mathematical models, machine learning-based models, statistical models, and hybrid methods. Boolean operators (AND, OR) and wildcards were used to ensure a broad yet focused retrieval of studies relevant to avian influenza modeling. Additionally, no restrictions were placed on publication dates, allowing the review to capture both foundational studies and the most recent advances in the field.

### 2.2 Screening

In the Screening stage, the collected articles underwent a rigorous initial review based on their titles and abstracts. This stage aimed to filter out irrelevant studies or those that did not focus on modeling approaches for avian influenza. Studies that appeared to meet the preliminary inclusion criteria were subjected to full-text review.

During the screening process, predefined inclusion and exclusion criteria were applied to ensure that only the most relevant studies were selected.

The filtering criteria applied during the Screening process were as follows: i) inclusion of studies that have been peer-reviewed, ii) inclusion of studies focused on avian influenza models, iii) inclusion of mathematical models, statistical models, machine learning-based models, or a combination thereof, and iv) exclusion of articles not written in English.

The screening was conducted independently by two researchers (S.H. and N.L.B.). If there was any uncertainty or disagreement regarding the inclusion of a study, the two researchers discussed the issue to reach a consensus, ensuring a more rigorous and objective selection process. A third researcher (J.D.K.) acted as the final referee.

### 2.3 Eligibility

In the eligibility stage, articles that passed the screening process were subjected to full-text review to confirm their relevance and quality. Each study was evaluated based on its contribution to the modeling of avian influenza, the methodology employed, and the robustness of the model validation and performance metrics. The assessment focused on whether the study provided a clear and detailed description of its modeling framework, including the parameter estimation and the datasets utilized. Studies that lacked methodological transparency or provided incomplete descriptions of their models were excluded from the final data extraction and synthesis.

### 2.4 Data extraction and synthesis

Following the final selection of eligible studies, a thorough data extraction process was conducted. A structured extraction form was used to systematically gather key information from each study. This included: i) type of model (categorizing the study into mathematical models (i.e. compartmental(ized) models), statistical models (i.e., regression models), machine learning models (i.e., neural networks, decision trees), or hybrid models (a combination thereof), ii) model parameters (details about the parameters used in the models, such as environmental factors, transmission rates, or population data), iii) validation methods (information on how the models were validated, such as through the use of external datasets, cross-validation, confusion matrices, or sensitivity analyses), iv) datasets (information about the datasets that the models had been used for training), and v) model outcomes (key results reported in the study, such as predictive accuracy, transmission dynamics, or intervention effectiveness).

The extracted data was then synthesized to compare and contrast different modeling approaches, highlight their strengths and limitations, and identify trends in the dynamics of avian influenza.

## 3 Results

**Fig. 1:**
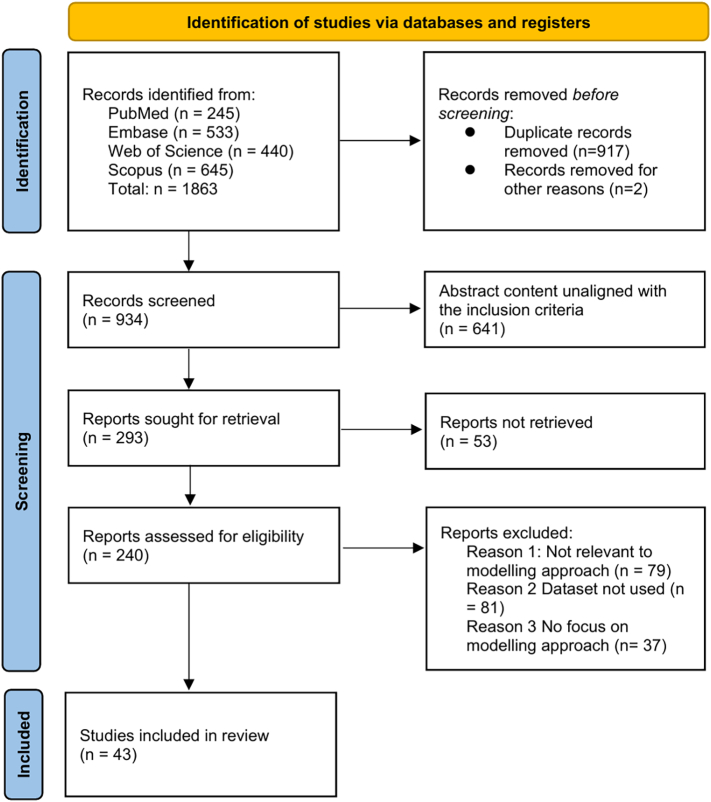
Flowchart of PRISMA.

The study identification process is depicted in Fig. 1. The reviewed literature was categorized into the following primary modeling approaches for avian influenza: mathematical models, statistical models, machine learning-based models, and hybrid models that integrate the previous methodologies. These models were further classified based on their specific applications, including risk assessment, outbreak prediction (such as hotspot detection), dynamic modeling, and parameter estimation models.

In this paper, mathematical models typically refer to Ordinary Differential Equations (ODEs) or Partial Differential Equations (PDEs). These models use parameters such as infection and recovery rates, while statistical models study the impact of environmental factors. These parameters are typically derived from data sources and these types of modeling offer insights into disease dynamics [9]. In contrast, machine learning-based models represent the majority of data-driven approaches, including Support Vector Machines (SVMs), Random Forests (RFs), Linear Regression, Decision Trees, and neural networks. These models use large datasets to predict hotspots or classify the presence of avian influenza based on various environmental and biological factors. Unlike mathematical models, they generally do not rely on pre-specified disease-related parameters, but instead identify patterns and correlations within data. Hybrid (or integrative) models represent a convergence of these methodologies. These models leverage the strengths of previous techniques—capturing the dynamic aspects of the disease through mathematical or statistical formulations while enhancing predictive capabilities through machine learning. The choice of model components depends on the specific application and available data.

### 3.1 Mathematical and statistical models

Mathematical models are essential tools in infectious disease epidemiology, enabling researchers to simulate the transmission dynamics of avian influenza, assess the impact of control measures, and estimate key epidemiological parameters. These models provide a structured approach to understanding how avian influenza spreads within and between bird populations, as well as its potential spillover to humans.

Among studies focusing on the mathematical modeling of avian influenza, 59.09 % employ compartmental or spatial models [10] to describe the virus's transmission patterns. Compartmental models, such as the Susceptible-Infected-Recovered (SIR [11,12]) framework and its variants, are widely used to simulate disease spread within bird populations. For instance, a SEEIRR model was applied to study H9N2 transmission in a Bangladeshi live bird market [13]. This model incorporated direct and environmental transmission pathways, offering insights into viral persistence and amplification. Such models help quantify critical parameters, including transmission and recovery rates, while providing an interpretable structure for understanding disease progression [14].

Beyond deterministic models, stochastic elements are often introduced to account for variability and uncertainty in real-world avian influenza transmission. Stochastic models incorporate random variations into the system, making them more reflective of the complexity of actual outbreaks [15,16]. One method of incorporating stochasticity is by extending classical frameworks such as the SEIR model. In stochastic versions, additional terms represent random fluctuations in transmission, recovery, maturity [15], or environmental factors, allowing for more nuanced predictions. For example, a stochastic model was developed to infer within-flock transmission dynamics in France, incorporating epidemiological uncertainty to enhance accuracy [17]. Since avian influenza transmission is influenced by multiple ecological and anthropogenic factors—including spatial patterns [[18], [19], [20], [21]], environmental conditions [22], and human activities [23]—these enhancements improve model realism and predictive power.

In addition to compartmental and stochastic models, statistical models play a crucial role in understanding and predicting AI outbreaks. These models rely on probabilistic frameworks and real-world data to estimate transmission dynamics, outbreak probabilities, and the effectiveness of control measures [[24], [25], [26]]. For example, a statistical transmission model was used to study the 2006 H5N1 outbreak in India, incorporating factors such as poultry trade, environmental conditions, and control measures [27]. Sensitivity analyses are frequently conducted in compartmental models to assess how variations in key parameters—such as latent periods or transmission rates—affect model outcomes [24]. This process helps evaluate model robustness and identifies which factors most significantly drive disease spread in different ecological settings. Numerical simulations further validate models by comparing predicted outcomes with real-world data, offering valuable insights into the virus's behavior under varying conditions.

Beyond studying transmission dynamics, mathematical models can be used for simulating control policies [28]. These models allow researchers to manipulate parameters and evaluate the potential effects of various intervention strategies, such as vaccination campaigns, culling policies, and movement restrictions. By adjusting these control variables, researchers can simulate different policy scenarios and assess their effectiveness in containing or mitigating avian influenza outbreaks. This simulation-based approach provides a quantitative framework for policymakers to make evidence-based decisions before implementing control measures in the field, ultimately aiding in risk assessment and outbreak management.

### 3.2 Machine learning models

Machine learning models offer a data-driven approach to understanding avian influenza outbreaks. Unlike mathematical models, which rely on predefined equations and assumptions, they learn patterns from large datasets, making them highly effective for predicting disease outbreaks and assessing risk levels. These models can process high-dimensional data, including environmental conditions [29] and historical outbreak records [30], to generate informed predictions.

Several studies (27.91 %) have employed machine learning models such as SVMs, RFs [30], Regression Models [31], and Gradient Boosting (XGBoost) [32]. These models typically learn from labeled training data, and therefore require datasets with known avian influenza cases to build predictive models.

For example, in a study on avian influenza outbreaks in the Netherlands, researchers used a Gradient Boosting model to analyze how wild bird densities and landscape features influence the spatial distribution of High-Pathogenic Avian Influenza (HPAI) outbreaks. The model successfully identified high-risk areas based on environmental variables such as wetland density, poultry farm locations, and migratory bird activity, demonstrating the effectiveness of machine learning in capturing complex ecological interactions [33]. This is particularly significant as it allows health authorities to focus surveillance and preventive measures on specific geographic areas, reducing unnecessary resource allocation and improving early detection.

In addition to traditional machine learning models, deep learning approaches such as the Multilayer Perceptron (MLP) method and Artificial Neural Networks (ANNs) have been explored for avian influenza prediction. A study conducted in China from 2013 to 2017 developed a neural network model to simulate the risk of H7N9 outbreaks in humans by integrating epidemiological, climatic, and demographic factors [34]. The model successfully predicted high-risk periods with remarkable accuracy, highlighting the potential of deep learning for real-time outbreak forecasting.

Similarly, a study in Korea applied an MLP-based deep learning model to assess the risk of HPAI outbreaks in poultry farms [35]. The model utilized KAHIS vehicle records and farm data to predict HPAI risks and was validated using data from the 2017/2018 epidemic for preemptive control [32].

Deep learning models are particularly powerful because they can automatically extract essential features from large and complex datasets, such as historical time series data. This makes them highly adaptable to evolving disease threats [35].

### 3.3 Hybrid models

Hybrid models, which combine mathematical, statistical, and machine learning approaches, have been explored in avian influenza research but remain relatively uncommon, accounting for only 5 out of 43 studies in this review (11.63 %). Unlike standalone mathematical or machine learning models, hybrid models do not follow a standardized framework; instead, their implementation varies significantly depending on the specific research objectives and available datasets. This variability underscores flexibility and complexity of hybrid modeling in avian influenza studies.

Typically, hybrid models integrate a mathematical framework, such as the Susceptible-Exposed-Infectious-Recovered (SEIR) model or its variants, with machine learning techniques, including agent-based models [36], neural networks [37], or other data-driven algorithms. This combination allows researchers to retain the mechanistic interpretability of mathematical models while leveraging the predictive power and adaptability of machine learning approaches. However, the specific role and implementation of each component depends heavily on the research focus—some studies use hybrid models primarily to enhance prediction accuracy [38], while others emphasize a more detailed understanding of transmission dynamics [37].

Beyond compartmental models, some researchers integrate statistical models, such as time-series approaches. A notable example is the Seasonal Auto-Regressive (SAR) model, which was incorporated into a hybrid SAR-Support Vector Regression (SVR) approach proposed by Zhang et al. for predicting avian influenza outbreaks [39]. This method combines SAR with SVR to effectively capture complex seasonal dynamics in epidemic data, significantly improving prediction accuracy compared to traditional models [39].

The mathematical component of hybrid models typically simulates the transmission dynamics of the virus, capturing its progression through compartments such as susceptible, exposed, infectious, and recovered populations [36]. These models are particularly valuable for analyzing the biological mechanisms underlying avian influenza outbreaks, as they incorporate key epidemiological parameters such as infection rates, recovery rates, and intervention effects (e.g., vaccination, culling, or movement restrictions). Additionally, stochastic elements can be introduced to account for randomness and uncertainty in real-world outbreak scenarios. In parallel, statistical approaches like time-series models (e.g., the SAR model) are often integrated into hybrid frameworks to enhance predictive capabilities. While compartmental models simulate disease spread based on theoretical assumptions, statistical models focus on identifying historical patterns and seasonal trends in outbreak data.

Meanwhile, the machine learning component enhances the model's adaptability and predictive capability by incorporating large-scale, real-world data. Machine learning algorithms are particularly effective at capturing complex, non-linear relationships that are often difficult to express mathematically. For example, agent-based models have been incorporated into hybrid frameworks to simulate individual behaviors and interactions within bird or human populations, offering a granular view of avian influenza spread in different environmental and social contexts [36]. By modeling the movement and interaction patterns of individual agents, hybrid models become more responsive to external factors such as climate conditions, migratory patterns, and human intervention strategies [40].

A key distinction between machine learning and mathematical/statistical model parameters in hybrid models is their dependence on datasets. In mathematical/statistical models, parameters are often fixed or derived from epidemiological studies, such as basic reproduction numbers (R₀) or transmission rates [41]. In contrast, machine learning models extract features from training data, meaning that their parameters can vary based on the dataset used. This distinction highlights the challenge of integrating both approaches, as their assumptions, data requirements, and parameterization methods differ.

### 3.4 Applications of models

To better understand the diverse applications of modeling approaches in avian influenza research, we categorized the collected studies into four primary subtypes: dynamic models, risk assessment models, outbreak prediction models, and parameter estimation models. This classification was based on the core objective of each study, ensuring a systematic analysis of their contributions.

Mathematical models dominate in dynamics modeling (50 %, *n* = 13) and parameter estimation (23.08 %, *n* = 6), highlighting their role in understanding disease transmission and refining epidemiological parameters. Machine learning models, in contrast, are widely used for risk assessment (50 %, n = 6) and outbreak prediction (41.67 %, *n* = 5), leveraging predictive analytics and spatial risk evaluation. Hybrid models, though representing only 11.36 % of total studies, integrate traditional and data-driven approaches, primarily for dynamics (40 %, *n* = 2) and outbreak prediction modeling (40 %, n = 2).

As shown in Table 1, the distribution suggests that while mathematical models remain central to disease modeling, machine learning is gaining traction in outbreak forecasting and risk assessment. The emergence of hybrid models reflects an increasing interest in combining traditional epidemiological methods with machine learning for a more comprehensive approach.

**Table 1:** Distribution of modeling approaches.

Model Type	Mathematical and Statistical Models (n, %)	Hybrid Models (n, %)	Machine Learning Models (n, %)
Dynamics Models	13 (50.0 %)	2 (40.0 %)	0 (0.0 %)
Risk Assessment Models	7 (26.92 %)	1 (20.0 %)	6 (50 %)
Outbreak Prediction Models	0 (0.0 %)	2 (40.0 %)	5 (41.67 %)
Parameter Estimation Models	6 (23.08 %)	0 (0.00 %)	1 (8.33 %)

**Fig. 2:**
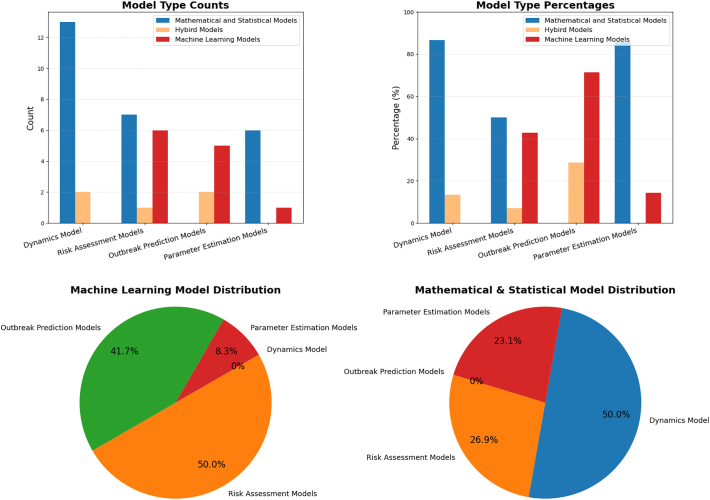
Distributions of models by objectives.

The distributions are displayed in Fig. 2.

## 4 Discussion

### 4.1 Datasets used in Avian Influenza modeling

The datasets used in avian influenza modeling are often region-specific, with studies focusing on particular countries such as Korea [31,42], China [34], Thailand [43], Japan [44], and Indonesia [45], as well as smaller regions like Georgia counties, NYC in the United States [23,46] and Jiangsu Province in China [47]. These datasets are typically highly localized, containing detailed information tailored to the unique epidemiological context of each study area. Mathematical and statistical models, in particular, rely on outbreak data specific to these regions, incorporating metrics such as infection rates, mortality data for specific bird species, and other regionally relevant transmission factors [38]. This approach allows mathematical models to simulate transmission dynamics with high accuracy within a confined area, making the results more applicable to specific localities. Machine learning models, in contrast, integrate a broader range of data types to capture a more comprehensive representation of avian influenza dynamics. These models combine traditional outbreak data with additional sources, such as climate factors (e.g., temperature, humidity) and agricultural practices. This diversity enables machine learning models to detect complex, non-linear relationships that may influence disease transmission across different regions. For instance, models that incorporate migratory data alongside local climate conditions provide deeper insights into when and where outbreaks may occur, allowing predictions that extend beyond localized contexts and enhancing broader surveillance efforts [29]. Although both modeling approaches benefit from outbreak-specific data, certain limitations remain. The reliance on localized datasets, particularly in mathematical and statistical models, can reduce the generalizability of findings to other regions or strains of the virus. Machine learning models, while capable of handling diverse data inputs, require high-quality, comprehensive datasets, which can be difficult to obtain consistently across regions. Additionally, both modeling approaches often depend on short-term or outbreak-specific data, limiting their ability to capture long-term trends and evolving environmental influences. Addressing these challenges through improved data-sharing initiatives and standardized data collection could enhance the robustness and applicability of avian influenza prediction and control models.

### 4.2 Validation methods in Avian Influenza models

Validation methods are essential for assessing the reliability of avian influenza models, but their application and reporting vary widely across mathematical, statistical, machine learning, and hybrid models [48]. Among mathematical models, approximately 25.58 % do not explicitly specify the validation methods used, creating gaps in understanding how well these models are fine-tuned or generalized beyond the study data. In such cases, model performance is typically inferred solely from the presented results, without a direct evaluation of robustness. For mathematical models that do report validation techniques, sensitivity analysis and numerical simulations are commonly applied [15,42,44,[49], [50], [51]]. Sensitivity analysis examines how variations in key parameters, such as infection rates or latent periods, influence model outcomes, providing insights into stability. Numerical simulations, meanwhile, test whether the model replicates theoretical expectations, ensuring internal consistency [46,49]. Statistical models often employ maximum likelihood estimation, adjusting parameters to maximize their fit to observed data [52], thereby improving alignment with real-world dynamics. Machine learning models generally adopt more explicit and quantitative validation methods. In prediction-focused studies, such as those forecasting outbreaks or identifying high-risk areas, F1-score [53] and confusion matrix [54] metrics are widely used. The F1-score balances precision and recall, making it particularly valuable for imbalanced datasets, while confusion matrices provide a detailed breakdown of prediction accuracy by distinguishing between true positives, false positives, true negatives, and false negatives. Some machine learning studies also use external datasets for validation to test whether models can generalize beyond the training data [55]. Hybrid models, which integrate mathematical and machine learning approaches, employ varied validation techniques based on their specific objectives and components. For example, predictive risk mapping models are often validated by comparing predicted high-risk areas with actual outbreak data over defined time periods to enhance both mathematical/statistical and machine learning performance [38]. Despite the diversity of validation methods, several challenges remain. Many mathematical and statistical models lack explicit validation protocols, limiting the transparency and reproducibility of their findings. Additionally, sensitivity analysis and numerical simulations, while useful for assessing internal consistency, often neglect external validation using real-world data. Machine learning models, though more standardized in their validation methods, rely heavily on F1-score-based evaluations, which may not fully capture the complexity of avian influenza dynamics. Furthermore, while external datasets significantly enhance model robustness, their use remains relatively limited, particularly in hybrid models. Addressing these gaps by adopting standardized validation methods and incorporating diverse datasets can improve the reliability and applicability of avian influenza models across different regions and scenarios.

### 4.3 Implications for policy and practice

While these modeling approaches vary in scope and validation, their outputs can be translated into actionable guidance for policymakers. Machine learning models, most often applied to risk assessment and outbreak prediction, have demonstrated strong predictive performance [56]; for example, Random Forest and gradient boosting approaches have been successfully used to forecast high-risk areas and anticipate outbreaks in advance [30]. Such models can support early warning systems and guide the allocation of surveillance resources to settings such as live bird markets and wetland–poultry interfaces. Mathematical and statistical models, with many focusing on transmission dynamics, remain essential for evaluating the impact of interventions. Prior studies have used compartmental frameworks to test alternative strategies such as vaccination, targeted culling, and movement restrictions, consistently showing that the timing and intensity of interventions strongly influence outbreak outcomes [10,30,57]. Hybrid models, though less common, have combined mechanistic insight with machine learning flexibility—for instance, SAR-SVR and agent-based hybrid approaches have improved predictive accuracy and enabled scenario planning under uncertainty [37,39,58,59].

However, validation practices remain inconsistent: approximately one-quarter of mathematical/statistical studies did not include explicit validation. This inconsistency underscores the need for standardized validation frameworks, transparent reporting of model assumptions and uncertainties, and third-party evaluation of predictive tools to enhance confidence in model outputs and ensure they can inform decision-making reliably. Furthermore, reliance on localized datasets limits generalizability. Strengthening cross-border data sharing platforms, harmonizing surveillance protocols across regions, and incorporating environmental and real-time epidemiological data are critical to developing robust and transferable models capable of guiding outbreak management at national and global scales. Adoption of these practices will enable policymakers to leverage modeling evidence more effectively for preparedness and evidence-based response during future avian influenza outbreaks.

## 5 Future directions for Avian Influenza modeling

To improve the accuracy, robustness, and applicability of avian influenza models, future research should prioritize several key areas. These include refining validation methods, integrating empirical data, broadening the scope of modeling, leveraging advanced techniques, and incorporating environmental factors. Each of these focus areas is explored in the following subsections.

### 5.1 Enhanced validation methods

Validation methods play a critical role in ensuring the reliability and robustness of avian influenza models. Future studies should implement multiple validation techniques, including cross-validation, bootstrapping, and external validation with real-world outbreak datasets. Utilizing diverse datasets spanning different regions and timeframes can enhance model accuracy and generalizability. Moreover, comprehensive reporting of validation protocols will improve transparency, enabling researchers to better assess and compare model performance [60]. Strengthening validation practices is essential for developing more reliable tools for outbreak prediction and intervention planning.

### 5.2 Integration of climate, environmental, and empirical data

Integrating climate, environmental, and empirical data into avian influenza models is crucial for understanding the factors driving disease transmission and improving model responsiveness to emerging outbreaks [61]. Climate variables such as temperature, humidity, and habitat changes significantly influence the spread of avian influenza, while real-time empirical data from health organizations—such as surveillance records and outbreak reports—provide a dynamic foundation for predictive modeling [62]. Developing eco-epidemiological models that account for interactions between wildlife, environmental conditions, and disease dynamics can offer a more comprehensive framework for predicting and managing outbreaks. These models can also help assess the impact of climate change on future transmission patterns, enabling more proactive public health interventions. Additionally, fostering data sharing and collaboration across institutions can further enrich datasets, improving both model training and validation. By integrating diverse data sources, researchers can develop models with greater predictive accuracy and real-world applicability. This holistic approach not only deepens the understanding of avian influenza dynamics but also enhances the capacity of models to generate actionable insights for outbreak prediction and control.

### 5.3 Building high-quality datasets

Developing high-quality datasets is essential for advancing avian influenza research, as well-structured and comprehensive data significantly enhance model accuracy and predictive capabilities. Studies have shown that integrating diverse datasets, such as topographic maps [63] and airline data, improves the modeling of avian influenza transmission dynamics by capturing key environmental and human-mediated factors that influence disease spread. These enriched datasets enable researchers to develop sophisticated eco-epidemiological models that incorporate climate, environmental, and empirical data, leading to more accurate outbreak predictions and response strategies. Moreover, fostering data-sharing initiatives and cross-institutional collaboration can improve dataset quality, strengthening model validation and generalizability. By leveraging a wide range of data sources, researchers can build robust, high-resolution models that offer actionable insights, ultimately enhancing the management and control of avian influenza outbreaks.

### 5.4 Computational costs in modeling

Computational cost is a crucial yet often overlooked factor in avian influenza modeling, particularly when considering the feasibility of real-time applications. Mathematical and statistical models, especially those involving large-scale numerical simulations or stochastic components, can require substantial computational resources when scaled to multi-regional or global levels. Machine learning models, particularly deep learning techniques, rely on high-dimensional datasets and complex algorithms, leading to increased training time and energy consumption. Hybrid models, which integrate both approaches, often amplify these computational demands due to the need to process diverse data sources and frameworks simultaneously. To address these challenges, future studies should focus on enhancing computational efficiency through dimensionality reduction (e.g., PCA [64]), parallel computing, and model pruning, while leveraging scalable, cloud-based, or high-performance computing systems [65]. Additionally, consistently reporting computational metrics such as runtime, memory usage, and energy consumption will improve the transparency and comparability of modeling approaches. These strategies will support the development of more efficient and accurate tools for outbreak prediction and response, making avian influenza models more applicable for real-time decision-making.

## 6 Conclusion

This systematic review has surveyed the diverse modeling approaches used to study avian influenza, categorizing them into mathematical, statistical, machine learning, and hybrid models. These models play a crucial role in understanding disease dynamics, predicting outbreaks, and evaluating intervention strategies. Mathematical/statistical models can be instrumental in capturing the transmission dynamics of avian influenza, while machine learning models excel at integrating complex datasets for predictive tasks. Hybrid models combine the strengths of both approaches, offering comprehensive tools for both understanding and forecasting.

Despite their potential, significant gaps remain in areas such as validation methods, computational efficiency, and the generalizability of models across regions and scenarios. Many studies rely on localized datasets, limiting broader applicability, while a lack of standardized validation protocols hinders comparability and transparency. To address these issues, future research should prioritize the integration of diverse datasets within a One Health framework, that is, recognizing the interconnectedness of human, animal (especially poultry and wild birds), and environmental health. For avian influenza, this entails combining climate and environmental indicators (e.g., temperature, migratory patterns, land use), veterinary surveillance data (e.g., poultry farm outbreaks, wild bird reservoirs), and real-time human epidemiological data to build more holistic and predictive models. Moreover, adopting more rigorous validation methods will be critical to ensure model robustness. Finally, careful consideration of computational costs and scalability will be essential for the deployment of practical, real-time tools to support outbreak monitoring and response.

By focusing on these improvements, the field of avian influenza modeling can advance toward more accurate, reliable, and globally applicable tools. These efforts will not only enhance our understanding of avian influenza dynamics but also support policymakers and public health professionals in making data-driven decisions to mitigate the impacts of future outbreaks.

## CRediT authorship contribution statement

**Shixun Huang:** Writing – review & editing, Writing – original draft, Visualization, Validation, Software, Resources, Methodology, Investigation, Formal analysis, Data curation, Conceptualization. **Nicola Luigi Bragazzi:** Writing – review & editing, Writing – original draft, Visualization, Validation, Supervision, Software, Resources, Methodology, Investigation, Formal analysis, Data curation, Conceptualization. **Zahra Movahedi Nia:** Writing – review & editing, Validation, Formal analysis, Conceptualization. **Murray Gillies:** Writing – review & editing, Validation, Resources, Formal analysis, Conceptualization. **Emma Gardner:** Writing – review & editing, Validation, Resources, Formal analysis, Conceptualization. **Doris Leung:** Writing – review & editing, Validation, Resources, Formal analysis, Conceptualization. **Itlala Gizo:** Writing – review & editing, Validation, Resources, Formal analysis, Conceptualization. **Jude D. Kong:** Writing – review & editing, Writing – original draft, Visualization, Validation, Supervision, Software, Resources, Project administration, Methodology, Investigation, Funding acquisition, Formal analysis, Data curation, Conceptualization.

## Funding sources

This research is funded by the NSERC Discovery Grant (Grant No. RGPIN-2022-04559) and the University of Toronto Undergraduate Work Study Program (which provided funding and opportunities for undergraduate student Shixun Huang). JDK acknowledges support from the NSERC Discovery Launch Supplement (Grant No. DGECR-2022-00454), the SSHRC New Frontiers in Research Fund – Exploratory (Grant No. NFRFE-2021-00879), Canada's International Development Research Centre (IDRC) (Grant No. 109981), as well as IDRC and the Foreign, Commonwealth and Development Office (FCDO) (Grant No. 110554–001) and Canada Research Chair in Community-Oriented Artificial Intelligence and Mathematical Modelling of Infectious Diseases (Award ID: CRC-2023-00234).

## Declaration of competing interest

The authors declare that they have no known competing financial interests or personal relationships that could have appeared to influence the work reported in this paper.

## Figures and Tables

**Table:** 

Study	Validation Methods	Datasets
Guinat et al. 2022 [17]	Key parameters were estimated from mortality data using ABC-SMC.	Duck death records in France
Kane et al. 2014 [30]	Buffer radii were varied as a sensitivity analysis to assess the effective range of farm influence on HPAI H5 outbreaks.	H5N1 outbreak incidence data in Egypt were sourced from the EMPRES-I system
Shih et al. 2022 [10]	Buffer radii were varied as a sensitivity analysis to assess the effective range of farm influence on HPAI H5 outbreaks.	HPAI H5 outbreak data (2012–2017) in Taiwan, including onset dates, GPS locations, and affected bird species
Pinotti et al. 2024 [13]	Parameters were inferred using a Bayesian MCMC approach.	Chicken data from an LBM in Chattogram, Bangladesh.
Yoon et al. 2020 [35]	Four metrics were used, including precision, recall, and F1-score	Data included all livestock and animal health records in Korea, plus visit logs from confirmed HPAI outbreak farms and related farms
Zhang et al. 2022 [47]	Time-dependent AUC was defined as the concordance probability within a fixed window ∆t. A 3-fold cross-validation repeated 10 times ensured prediction stability	Clinical and lab data of 96 H7N9 patients hospitalized in Jiangsu (Jan 2016–May 2017) were retrospectively analyzed
Lu et al. 2010 [39]	Model adequacy was verified through residual analysis of prediction errors on the test dataset	Verified H5 and H7 outbreak reports (Dec 2003–present) from various countries were obtained from the OIE website
Rorres et al. 2011 [16]	Optimal parameters were obtained by minimizing the formula expression	Data from the 1983–84 avian influenza epidemic in Pennsylvania were used
Lang 2024 [63]	Results were compared with real case data	Data included H7N9 cases (China CDC), maps (topography, transport, population), bird migration routes, and 2013 meteorological data (CMA)
Dong et al. 2022 [34]	Simulation accuracy was assessed	Data included H7N9 human cases (2013–2017), poultry, livestock value, market data, and vegetation index
Hayama et al. 2022 [15]	Sensitivity analysis and numerical simulation were performed.	HPAI outbreak data in Japan (2020−2021)
Kim et al. 2008 [42]	Sensitivity analysis and numerical simulation were performed.	Avian influenza outbreak data in South Korea, April 2008.
Le Menach et al. 2006 [57]	Model results were assessed via correlation and multivariate sensitivity analysis.	Focused on the 2003 A/H7N7 outbreak in Gelderland and Utrecht, where 185 farms were infected from Feb 28.
Putro et al. 2008 [59]	Not Specified	Data were sourced from Bandung City (Bandung Dalam Angka, 2005)
Penny et al. 2010 [11]	Akaike's Information Criterion (AIC) was performed.	Data included bird migration/wintering patterns and H5N1-positive dead waterbirds at Lake Constance in 2006.
Andronico et al. 2019 [58]	Sensitivity analysis and numerical simulation were performed.	Data covered 471 HPAI IPs, 461 culling dates, and poultry farms within 100 km in southwest France (2016–2017)
Yoon et al. 2005 [31]	Used Kaplan–Meier curves, adjusted R^2^, and standard error (SE) for evaluation.	Data from 10 HPAI-positive chicken farms during the Dec 2003–Mar 2004 Korea outbreak
Bos et al. 2007 [15]	Sensitivity analysis and numerical simulation were performed.	Data from 241 infected and depopulated farms during the 2003 Dutch AI epidemic
Ferguson et al. 2003 [50]	Sensitivity analysis and numerical simulation were performed.	Data Included sequence, phylogenetic, and epidemiological data.
Boni et al. 2009 [51]	Sensitivity analysis and numerical simulation were performed.	Data Included H1N1 and population data from Vietnam.
Dorea et al. 2010 [23]	Not Specified	Data were obtained from two counties in Georgia.
Nguyen et al. 2010 [36]	Not Specified	Data Included daily reports of poultry deaths in northern Vietnam, November 2005. [31]
Upadhyay et al.2008 [27]	Not Specified	Outbreak data from 16 countries during February 13–18, 2006.
Pop et al., 2008 [37]	Not Specified	Data from Cluj Napoca
Kuo et al. 2009 [19]	Not Specified	Monthly data (Jan 2004–Dec 2006) from Indonesia, Thailand, Vietnam, Cambodia, and China.
Rao, 2008 [12]	Not Specified	Data included January 2008 outbreak data from West Bengal.
Marquetoux et al. 2012 [43]	Not Specified	HPAI H5N1 outbreak data in Thailand since January 2004 (collected by DLD veterinarians).
Wang et al., 2012 [55]	Cross-validation assesses model performance and an independent external test set provides unbiased evaluation.	1777 AIVs of various subtype
Minh et al., 2011 [20]	Not Specified	HPAI H5N1 outbreaks in four Mekong Delta provinces, Vietnam (Jan–Mar 2009)
NICKBAKHSH et al., 2014 [56]	Not Specified	Poultry Network Database (PND), UK
Khazeni et al., 2009 [46]	Sensitivity analysis and numerical simulation were performed.	2009 H1N1 pandemic costs in NYC (2009 USD, GDP-deflator adjusted).
Vaidya and Wahl, 2015 [22]	Use F-tests for nested models; compare AICc values otherwise.	Data from Northern Europe
Tuncer and Martcheva, 2013 [45]	AICc for model comparison.	Poultry outbreaks data in Indonesia (2008–2011)
Mannelli et al., 2007 [25]	Negative-binomial deviance residuals (accounting for overdispersion)	poultry farm and HPAI case data (Lombardy, Veneto).
Hsu and Shih, 2010 [24]	Sensitivity analysis and numerical simulation were performed.	Transmission and control data of an emerging influenza pandemic in a small-world airline network
Stevens et al., 2013 [21]	ROC AUC, Sensitivity analysis	Human/poultry/water/road/rice data from CIESIN, Prosser et al., Van Boeckel et al., FAO.
Dong et al., 2015 [38]	Improved logistic regression (10-run averaged) vs. traditional model risk maps validated on 101 H7N9 cases (2014).	Data from age/sex distribution in 460 H7N9 human cases (China, 2013–2014).
Liu and Fang, 2015 [14]	Not Specified	Nationwide H7N9 surveillance data (animal/human).
Yang et al., 2015 [26]	Sensitivity analysis and numerical simulation were performed.	Household H7N9 cases/contacts in China: 125 (1st wave), 187 (2nd wave) across 10 provinces
Hill et al., 2017 [18]	MCMC simulation	Bangladesh H7N9 data: poultry census, case reports, risk factors (LBMs, ducks, water, rice), and human cases.
Huang et al., 2021 [29]	ROC, variable importance, correlation analysis	H7N9 cases (2013–2017, 27 provinces), meteorological data (temperature/precipitation), farming/poultry stats, and geospatial data (water distance, NDVI).
Liu et al., 2023 [32]	Grid Search Cross-Validation (64 % train, 36 % test), AUC, RR, PR	H9N2-infected laying hen production data (Oct–Dec 2012, 3 houses, China).
Schreuder et al., 2022 [33]	Acc, Recall, AUC-ROC, predicted risk maps	HPAI-linked bird density (winter counts 2012–2015) and land cover data (Netherlands).

## Data Availability

No data was used for the research described in the article.
